# Laboratory Notes on Poison in Sorghum1Read at the meeting of the Iowa-Nebraska Veterinary Association, Omaha, October 2, 1902.

**Published:** 1902-11

**Authors:** S. Avery

**Affiliations:** Chemist, Nebraska Experiment Station, Lincoln, Neb.


### LABORATORY NOTES ON POISON IN SORGHUM.[1]

By S. Avery,
CHEMIST, NEBRASKA EXPERIMENT STATION, LINCOLN, NEB.

The symptoms of sorghum-poisoning in cattle are too well known in the West to require description. The theories that have been formed to account for the fatal results that have followed the eating of the plant are very numerous. For a number of years the veterinarian of the Nebraska Experiment Station, Dr. A. T. Peters, has investigated the various outbreaks, and some time ago arrived at the following conclusions:

1. There is no foundation for the theory that the animals choke to death.

2. There is no foundation for the theory that the animals die from indigestion.

3. Animals die from the effect of a poison which has been elaborated in sorghum of stunted growth.

4. The symptoms are those of prussic acid.

These observations served as a starting-point for the work of the chemist. About the first of last August Mr. Henry B. Slade, formerly assistant chemist to the Nebraska Station, now chemist of the Idaho Station, discovered that prussic acid could be obtained from the stalks of sorghum taken from a field of poisonous cane. Mr. Slade also observed that there is evidence that the prussic acid is present as a glucoside and is liberated by an enzyme. On the 26th of August the writer of this paper took up the work. With the data at hand it has been comparatively easy to move rapidly toward a solution of the entire problem.

If green sorghum leaves (say 100 grammes) be ground up, placed in a flask with three times the weight of water, and the mixture distilled, prussic acid may be detected in the distillate by any of the well-known chemical tests. I hold in my hand three tubes containing prussic acid derived from cane. The acid, to be sure, is not present as such, but has been converted into highly

[1] Read at the meeting of the Iowa-Nebraska Veterinary Association, Omaha, October 2, 1902.

colored derivatives. No. 1 is from a field near the station, which had escaped the frosts. The acid has been converted into ferric sulphocyanate. No. 2 represents acid from the same field converted into Prussian blue. These two samples were gathered September 30th. No. 3 represents a field from which several cattle had eaten, with fatal effect.

The writer has tested sorghum—big and little, young and old—and has never failed to find at least traces of prussic acid in the living leaf. Kaffir corn leaves also contain this poison; but other forage plants—clover, alfalfa, grasses, and corn—give no test for prussic acid. The attention of those who, in the past, have confounded sorghum- and kaffir corn-poisoning with indigestion and bloat is respectfully called to this observation.

About September 8th a careful examination of a field of healthy sorghum was made. The stalks stood eight feet high and the heads were well out. This field was estimated to contain thirty tons of green sorghum per acre. The amount of prussic acid present was about one pound per acre. This amount, distributed as it was through thirty tons, was well below the danger mark. A determination of the amount of prussic acid in some second-growth sorghum showed that the latter contained just twice the percentage of the above. This is also safe, but might become dangerous if we should have two or three weeks of warm, dry weather.

The prussic acid in sorghum and kaffir corn is found in the stems and leaves. It has not been detected in the seed in stalks as large as a lead pencil, or in roots. If sorghum of fair growth, about four feet high or higher, is ground up, stalks and all, and distilled, little or no prussic acid is found in the distillate. Now when cattle eat sorghum of stunted growth they consume not only feed abnormally high in poison, but exactly the part of the plant where all of the poison is found. On the other hand, sorghum of good growth offers to the animal leaves weaker in poison, palatable parts free from and probably more or less of an antidote for poison. Experiments carried on at the Nebraska Station show that prussic acid is much more active when administered by itself than when first mixed with grape-sugar.

The question as to the form in which prussic acid exists in the plant is still unanswered. A discussion of the data at hand would be of more interest to chemists than to veterinarians. It is sufficient to say the acid is not present as free acid, since the dried plant still may contain relatively large amounts. Samples kept for six weeks in the laboratory give strong tests for prussic acid. On the other hand, a sample ground up and moistened with water lost all of its prussic acid on drying.

If the writer may venture from the safe ground of experiment into the uncertain field of speculation, he would suggest that the presence of nitrates in the soil may facilitate the formation of prussic acid in the plants. It is well known that in semi-arid sections much of the nitrogen in the soil is in the form of nitrates, while in well-watered sections the greater part of the nitrogen is combined with humus. May not this fact explain why sorghum stunted by an occasional dry season is seldom fatal to stock in the East, and that many of the most severe outbreaks occur where irrigation is practised?

In conclusion, let me say that I trust that these laboratory notes of the chemist have fully confirmed the diagnosis of the veterinarian.

